# Identification and characterization of a maize-associated mastrevirus in China by deep sequencing small RNA populations

**DOI:** 10.1186/s12985-015-0384-3

**Published:** 2015-10-05

**Authors:** Sha Chen, Qingqing Huang, Liqi Wu, Yajuan Qian

**Affiliations:** State Key Laboratory of Rice Biology, Institute of Biotechnology, Zhejiang University, Hangzhou, People’s Republic of China

**Keywords:** Mastrevirus, Small RNA deep sequencing, ssDNA viruses, vsiRNAs

## Abstract

**Background:**

*Maize streak Reunion virus* (MSRV) is a member of the *Mastrevirus* genus in the family *Geminiviridae*. Of the diverse and increasing number of mastrevirus species found so far, only *Wheat dwarf virus* and *Sweetpotato symptomless virus* 1 have been discovered in China. Recently, a novel, unbiased approach based on deep sequencing of small interfering RNAs followed by *de novo* assembly of siRNA, has greatly offered opportunities for plant virus identification.

**Methods:**

Samples collected from maize leaves was deep sequencing for virus identification. Subsequently, the assay of PCR, rolling circle amplification and Southern blot were used to confirm the presence of a mastrevirus.

**Results:**

*Maize streak Reunion virus* Yunnan isolate (MSRV-[China:Yunnan 06:2014], abbreviated to MSRV-YN) was identified from maize collected from Yunnan Province, China, by small RNA deep sequencing. The complete genome of this virus was ascertained as 2,880 nucleotides long by conventional sequencing. A phylogenetic analysis showed it shared 96.3 % nucleotide sequence identity with the isolate of *Maize streak Reunion virus* from La Reunion Island. To our knowledge, this is the first identification of MSRV in China. Analyses of the viral derived small interfering RNAs (vsiRNAs) profile showed that the most abundant MSRV-YN vsiRNAs were 21, 22 and 24 nt long and biased for A and G at their 5’ terminal residue. There was a slightly higher representation of MSRV-YN siRNAs derived from the virion-sense strand genome than the complementary-sense strand genome. Moreover, MSRV-YN vsiRNAs were not uniformly distributed along the genome, and hotspots were detected in the movement protein and coat protein-coding region.

**Conclusions:**

A mastrevirus MSRV-YN collected in Yunnan Province, China, was identified by small RNA deep sequencing. This vsiRNAs profile derived from MSRV-YN was characterized, which might contribute to get an insight into the host RNA silencing defense induced by MSRV-YN, and provide guidelines on designing antiviral strategies using RNAi against MSRV-YN.

**Electronic supplementary material:**

The online version of this article (doi:10.1186/s12985-015-0384-3) contains supplementary material, which is available to authorized users.

## Introduction

Viruses in the family *Geminiviridae* are taxonomically classified into seven genera (*Begomovirus*, *Curtoviurs*, *Mastrevirus*, *Topocuvirus*, *Becurtovirus*, *Turncurtovirus*, *Eragrovirus*) based on insect vector, host range and genome organization [1]. According to their genome components, geminiviruses can be characterized into two groups: bipartite geminiviruses with two similar-sized single-stranded (ss) DNA genomes and monopartite geminiviruses with one ssDNA genome. The genus *Mastrevirus* contains species with a monopartite genome of approximately 2.7 kb encapsidated in geminate virions, which encode four proteins separated by two intergenic regions (large intergenic region [LIR] and small intergenic region [SIR]). The two proteins encoded on the virion-sense strand are the movement protein (MP), functioning in cell-to-cell movement, and the coat protein (CP), encapsidating the virion-sense ssDNA and acting as the nuclear shuttle protein (NSP) for viral DNA. The complementary-sense strand encodes the replication-associated protein Rep and Rep A [2]. The Rep is expressed through a transcription splicing mechanism of the transcripts for C1 and C2 [3], while Rep A is expressed via a transcript spanning the C1 ORF.

Members of *Mastrevirus* are mainly transmitted by leafhoppers and can infect a wide variety of monocotyledonous and dicotyledonous plants. Currently, there are 29 recognized members belonging to the genus *Mastrevirus* based on ICTV classification [2]. *Maize streak Reunion virus* (MSRV) is an emerging new member of genus *Mastrevirus* that has been reported only in La Reunion Island and Nigeria and shares less than 57 % genome-wide identity with all other known mastreviruses [4, 5]. Apart from *Maize streak virus* (MSV), MSRV is the only mastrevirus species detected in maize. Of the diverse and increasing number of *Mastrevirus* species found so far, only *Wheat dwarf virus* (WDV) and *Sweetpotato symptomless virus* 1 (SPSMV-1) have been discovered in China [6, 7].

Recently, a novel, unbiased approach for plant virus identification has been developed by deep sequencing and assembly of virus-derived small interfering (si) RNAs [8]. Upon virus infection, the host can initiate an efficient defense to ward off invading nucleic acids by cleaving viral double-stranded RNA or imperfectly folded viral self-complementary single-stranded RNA sequences using Dicer-like proteins (DCLs), which generates different classes of small RNAs (sRNAs). These viral siRNAs are overlapping in sequence and can be assembled into long contiguous fragments (contigs) mapping to the invading viral genome [9], which provides the theoretical basis for identifying viruses using small RNA deep sequencing. Unlike traditional genetic methods for virus diagnostics mainly relying on serological or molecular characterization, the biggest advantage of this approach is that it requires no a priori knowledge of the pathogen.

In this paper, we report the first identification using high-throughput sequencing of small RNAs populations of a DNA virus infecting maize in China. Further, the genome organization and a phylogenetic analysis suggest that the virus is a member of *Mastrevirus*, *Geminiviridae*, most closely related to MSRV isolated from La Reunion Island.

## Results and discussion

### De Novo assembly of small RNAs and the maize streak reunion virus YN isolate identified from maize

When a sample (named YM06) collected from leaf of field-grown maize plant in Yuanmou County, Yunnan Province, China, was sequenced using the Illumina Hiseq 2000 Solexa platform, 10,781,056 clean reads were produced after removing adaptor sequences and the low quality reads. A total 10,739,457 sRNA sequences of 18–28 nt were obtained and used to assemble contigs using the Velvet program by a *k*-mer value of 17, resulting in the identification of 536 contigs (Table 1). A BLASTn analysis of assembled contigs against the NCBI database, using a highly homology sequence search, identified three contigs showing identities of 96 to 100 % with different isolates of MSRV (Additional file 1: Table S2), which are members of genus *Mastrevirus, Geminiviridae*, suggesting the presence of a potential MSRV isolate in the analyzed sample.

**Table 1:** Deep sequencing data and assembly of small RNAs

Characteristic	Value
Reads after removing adaptor (clean reads)	10,781,056
18-28 nt reads	10,739,457
Total contigs after assembling small RNA	536
Contigs matching *Maize streak Reunion virus*	3
Reads matching *Maize streak Reunion virus-*YN genome (0 mismatch)	237,209

Primer pair F/R1 was designed based on the assembled viral contigs, and a nucleotide fragment of 720 bp was obtained. A BLASTn search with this fragment in the NCBI database showed that it shares high identity of 98 % to the isolate of MSRV (GenBank: JQ624880), with a query cover of 100 % (data not shown), further confirming the actual presence of the potential MSRV isolate. Thus, we tentatively named the isolate MSRV-[China:Yunnan 06:2014], which was abbreviated to MSRV-YN.

In a PCR using primer pair F/R1 to investigate the incidence of MSRV-YN among the 22 maize samples collected from Yuanmou county, Yunnan province, amplicons of the expected size, about 700 bp, were generated in ten of the 22 tested samples, revealing an infection rate for isolate MSRV-YN of 45.5 % (data not shown). To our knowledge, this is the first report of a isolate of MSRV in China.

### Full-length cloning, RCA and Southern blot detection of MSRV-YN

Back-to-back primers F/R designed based on the assembled contigs were used to amplify the full-length genomic sequences of MSRV-YN from a crude extract of total DNA from the maize sample, yielding a fragment of about 3.0 kb. This fragment was then cloned and sequenced by conventional Sanger sequencing and assembled by SeqMan (Lasergene package, Version 7.1.0), generating a complete sequence 2880 bp long (GenBank: KT717933). A BLASTn search using the completed genome of MSRV-YN in NCBI revealed significant similarity (98 %) to the isolate of MSRV from La Reunion Island (GenBank: JQ624880).

**Fig. 1:**
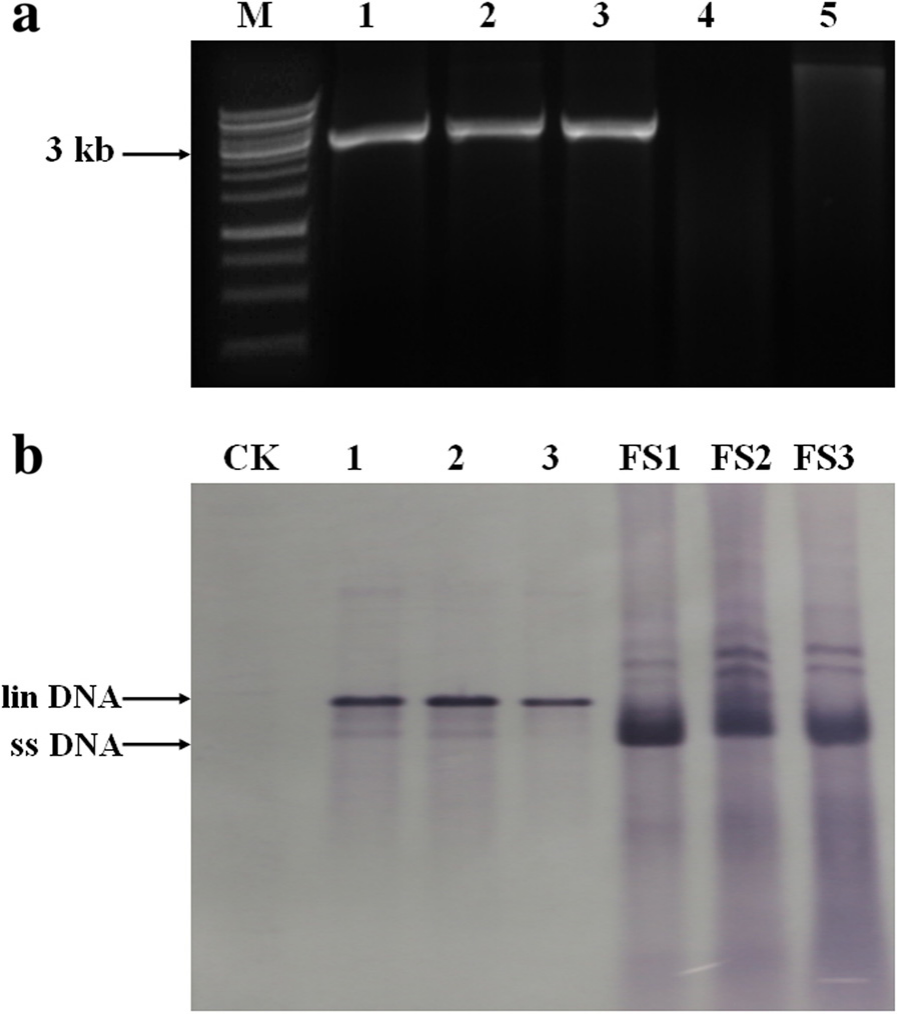
Detection of MSRV-YN by rolling circle amplification (RCA) and Southern blot. **a** Analyses of the RCA products from three randomly selected MSRV-YN-infected maize leaves samples (line 1 to 3) and health control (line 4), digested by BamHI restriction enzyme or were not digested (line 5). M, GeneRuler 1 kb DNA Ladder (SM0311, Thermo). **b** The digested RCA products using the BamHI restriction enzyme (line 1 to 3) and corresponding total DNA extracted from infected field samples (line FS1 to FS3) were further identified by Southern blot. CK, healthy control (maize leaves not infected by MSRV-YN). The positions of the linear (lin), and single-stranded (ss) DNA are indicated

To further prove the presence of MSRV-YN, the aforementioned DNA was used as template for RCA amplification followed by restriction fragment length polymorphism (RFLP) analyses, which is a nonsequence-specific method that potentially allows the identification of all circular DNA viruses. Three different RCA-amplified products from randomly selected MSRV-YN positive samples were digested with the BamHI restriction endonuclease, which proved to have a single restriction site based on the information of the obtained complete genome sequence of MSRV-YN. As expected, a single DNA fragment of about 3.0 kb was obtained, the size expected for a mastrevirus. Meanwhile, no band was ever found in the negative control from healthy maize leaves (Fig. 1a). Next, Southern blot analysis with an MSRV-YN-specific probe further confirmed the presence of the viral genome of MSRV-YN and validated the RCA products digested by BamHI, while the control from healthy maize leaves tested negative for MSRV-YN (Fig. 1b). All these data strongly confirmed the presence of a circular DNA virus provisionally named MSRV-YN in the maize samples.

### Genomic organization and sequence analysis of MSRV-YN

**Fig. 2:**
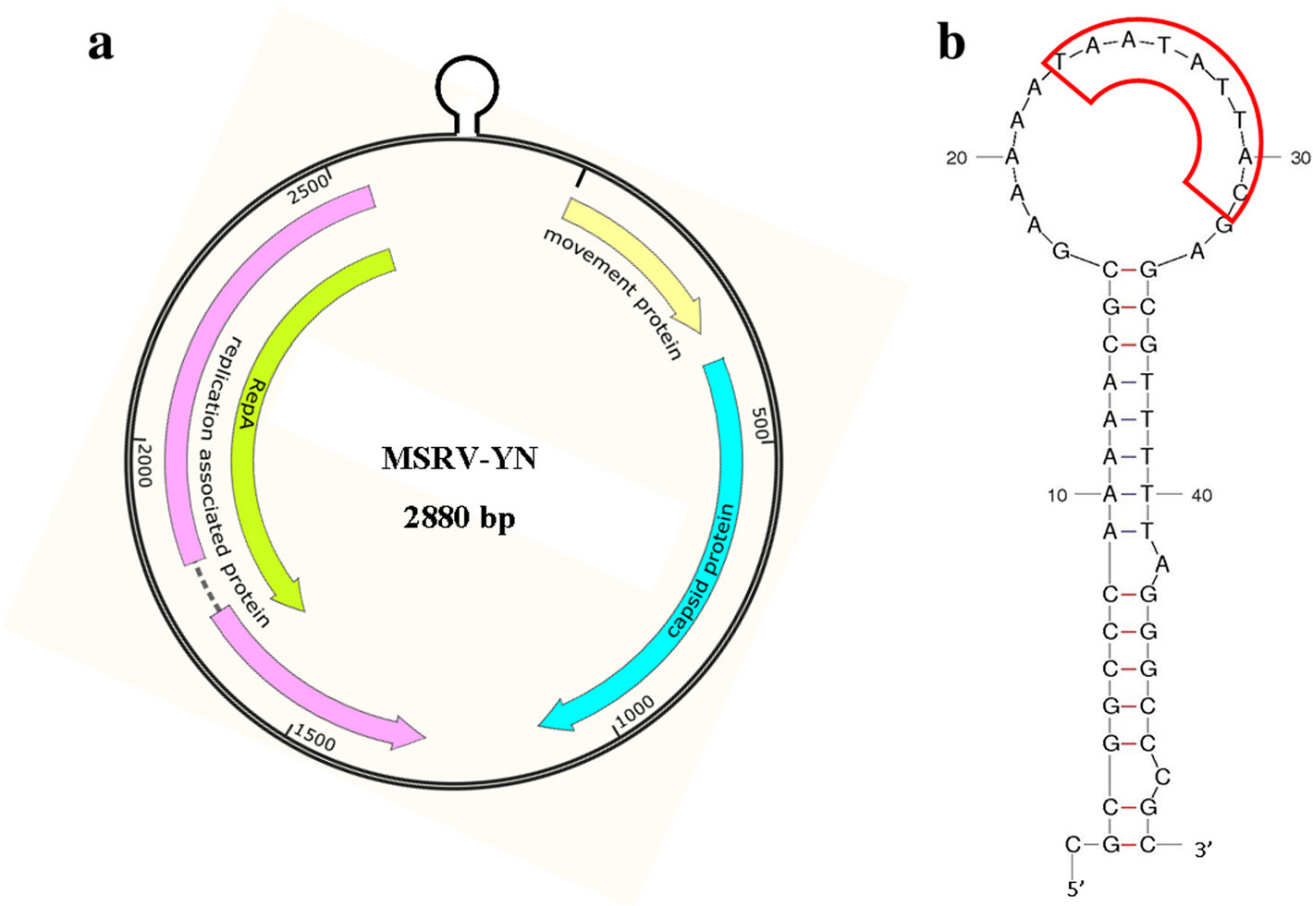
Schematic representation of the genome organization of the MSRV-YN with four predicted open reading frames (ORFs) (**a**) and the loop in the intergenic region shown on the top with the nonanucleotide sequence (TAATATTAC) highly conserved in almost all geminiviruses (**b**)

The ORF Finder within the Snap Gene software [10] indicated the presence of the four major open reading frames and two intergenic regions (IRs) in the MSRV-YN genomic sequence (Fig. 2a), which is a typical genomic organization of mastreviruses [11]. The virion-sense strand encodes a putative movement protein (MP) and a coat protein (CP), and the complementary-sense strand encodes two replication associated proteins (Rep and RepA), separated by a large and small IR (LIR, SIR) (Fig. 2a). As previously reported for almost all known geminiviruses, MSRV-YN contains the highly conserved nonanucleotide sequence TAATATTAC, which is within the loop of the LIR (Fig. 2b). On the other hand, the nucleotide and amino acid sequence alignments between MSRV-YN and other members of the *Mastrevirus*, revealed that MSRV-YN shares <63 % genome-wide pairwise identity with known *Mastrevirus* species, except that for a close identity with MSRV (GenBank: KJ437669); complete genome identity and amino acid identities for MP, CP, Rep, RepA are 96.3, 99.0, 98.0, 95.1 and 93.3 %, respectively (Table 2), indicating that MSRV-YN is a member of *Mastrevirus* and most closely related to MSRV.

**Table 2:** The identities (%) between MSRV-YN and other mastreviruses based on nucleotide and amino acid alignment

Virus	Abbreviation	GenBank accession	Genome size (nt)	Nucleotide identity (%)	Amino acid similarity (%)			
					MP	CP	Rep	RepA
*Wheat dwarf virus*	WDV	HF968638	2750	43.2	41.6	29.5	44.0	35.3
*Oat dwarf virus*	ODV	NC_010799	2740	43.0	37.8	29.9	42.3	34.2
*Eragrostis minor streak virus*	EMSV	NC_015553	2689	44.3	13.7	34.7	45.3	38.7
*Chickpea yellows mastrevirus*	CpYV	JN989439	2557	42.5	41.4	38.3	44.7	32.8
*Chickpea chlorosis virus*	CpCV	KC172700	2590	43.1	38.0	35.2	47.0	37.4
*Tobacco yellow dwarf virus*	TobYDV	NC_003822	2580	44.4	38.0	34.0	45.8	35.6
*Paspalum dilatatum striate mosaic virus*	PDSMV	NC_018576	2806	46.6	19.8	45.5	42.3	33.8
*Paspalum striate mosaic virus*	PSMV	NC_018530	2816	46.5	20.8	44.7	41.7	33.0
*Bromus catharticus striate mosaic virus*	BrSMV	NC_014822	2797	46.6	18.8	42.3	42.8	34.2
*Wheat dwarf India virus*	WDIV	KJ028209	2784	62.3	57.8	61.8	53.2	46.6
*Maize streak Reunion virus*	MSRV	KJ437669	2879	96.3	99.0	98.0	95.1	93.3

### Phylogenetic analysis

**Fig. 3:**
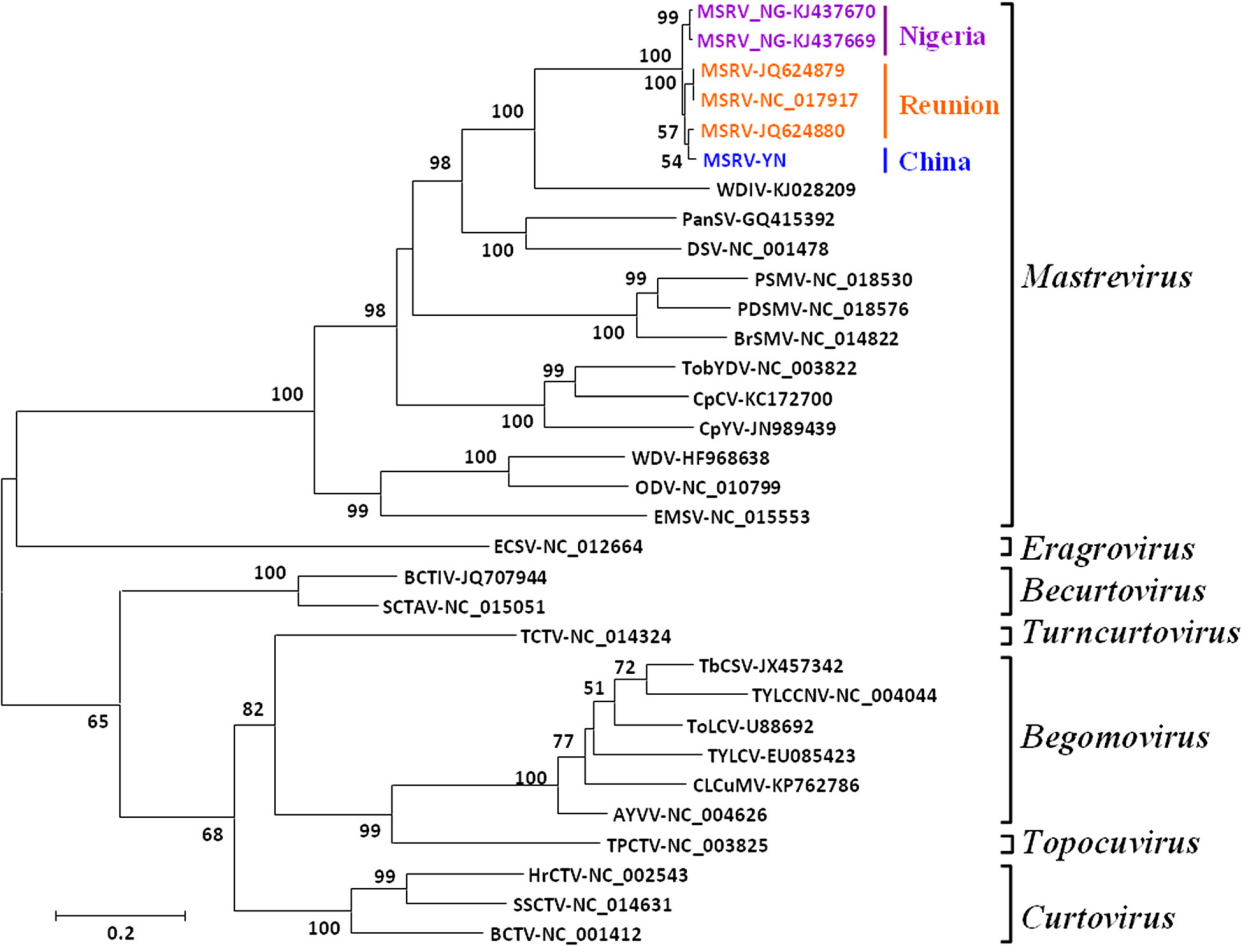
Phylogenetic analysis of nucleotide sequences for the whole genome of representative geminivirus members. The evolutionary history was inferred using the neighbor-joining method. The percentage of replicate trees in which the associated taxa clustered together in the bootstrap test (1000 replicates) is shown next to the branches. Evolutionary distances were computed using the maximum composite likelihood method and given as the number of base substitutions per site. The analysis involved 32 nucleotide sequences. All positions containing gaps and missing data were eliminated. Evolutionary analyses were done in MEGA6 [13]

A neighbor-joining tree based on full-genome nucleotide sequences of representative geminivirus members was constructed using Clustal W [12] in MEGA6 [13]. In accordance with the relationships predicted by sequence comparisons (Table 2), the MSRV-YN genome grouped perfectly with the known mastreviruses and clustered together with those reported isolates of MSRV (Fig. 3). Phylogenetic analyses further comfirmed that MSRV-YN is a member of genus *Mastrevirus* and most closely related to MSRV.

### Characterization of viral derived siRNAs from MSRV-YN

**Fig. 4:**
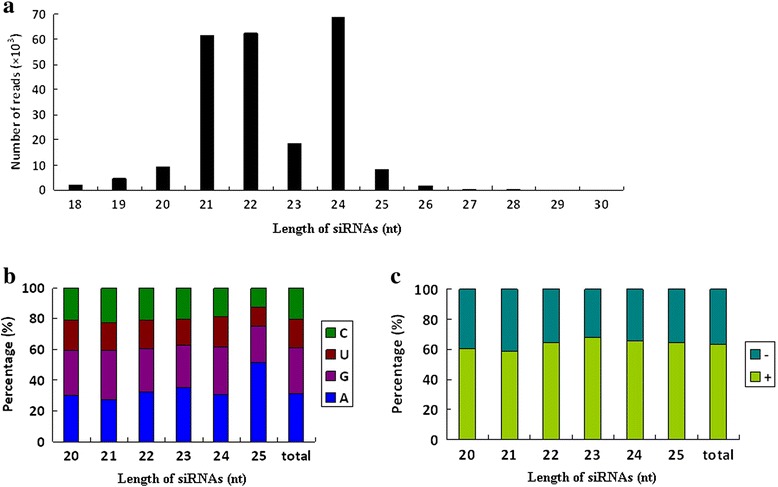
Characterization of MSRV-YN derived siRNAs. **a** Size distribution of total vsiRNAs for MSRV-YN. **b** Relative frequency of the four different 5’-terminal nucleotides in 20–25-nt vsiRNAs and total vsiRNAs. **c** Polarity distribution of the 20–25-nt vsiRNAs and total vsiRNAs that perfectly matched the virion sense strand (+) and complementary sense strand (−) genomic sequence. Histograms represent the ratios of total vsiRNAs within each category

The identification of viral small interfering RNAs (vsiRNAs) mapping to specific viral genome provides strong evidence for presence of the virus. We characterized the profile of vsiRNAs derived from MSRV-YN (Fig. 4). A total of 237,209 vsiRNAs were obtained using the short sequencing reads alignment software of Bowtie with zero mismatch (Table 1). The vsiRNAs population was dominated by species of 21, 22 and 24 nt, accounting respectively for 26.0, 26.4 and 28.9 % of the total vsiRNAs (Fig. 4a), suggesting the involvement of diverse DCLs in the biogenesis of MSRV-YN-derived vsiRNAs. Our observations were in line with previous reports describing the distribution of viral siRNA lengths for DNA viruses such as *Tomato yellow leaf curl China virus* (TYLCCNV) in *Nicotiana benthamiana* [14] and *Cabbage leaf curl virus* (CabLCV) in *Arabidopsis thaliana* [15].

Previous studies have indicated that the recruiting of sRNAs into specific AGO complexes mainly depends on the identity of the first 5’ -nucleotide of sRNAs [16, 17]. To deduce the potential interactions between MSRV-YN-derived siRNAs and distinct AGO complexes, the 5’ -nucleotide specificity in vsiRNAs of 20–25 nt was analyzed. The results revealed the prevalence of adenosine (A) and guanidine (G) compared with cytosine (C) and uridine (U) at the 5’ -terminal position of vsiRNAs (Fig. 4b). In *A. thaliana*, AGO2 and AGO4 predominantly favored small RNAs starting with the 5’ -terminal A, AGO1 preferentially recruited sRNAs initiating with a 5’ -terminal U, while AGO5 predominantly bound small RNAs that with a 5’ -terminal C [16, 17]. It is still unknown which of the remaining AGOs (if any) could preferentially bind sRNAs with a 5’ -terminal G. Our results suggested that the production of MSRV-YN-derived sRNAs preferentially involves AGO2 and AGO4, whereas the association with AGO1 was relatively lower. Notably, the 5’ -terminal residue bias of geminivirus-derived siRNAs presented very diverse results; 5’ -terminal residue biases for U and A were observed in CabLCV and TYLCCNV, whereas the 5’ -terminal residue C was preferred in TYLCSV [14, 15, 18]. In our case, a biased accumulation of 5’ -terminal G was observed (Fig. 4b), which is not very common in plants. In many cases reported, G was the least preferred base [14, 18, 19]. Although the biological meaning of these associations is not yet very clear, this finding might suggest the involvement of diverse AGOs in different plant species for vsiRNAs sorting.

When the vsiRNAs strand polarity was evaluated using the 20–25 nt vsiRNAs, a slightly higher representation of MSRV-YN siRNAs derived from the virion-sense strand (positive vsiRNAs) was unexpectedly observed, accounting for about 63.46 % of the total vsiRNAs, irrespective of length (Fig. 4c), suggesting that the different DCLs do not show strand biases. Strand preference can arise from cleavage of dsRNAs formed during replication for RNA viruses, bidirectional transcription for a circular viral DNA, or highly structured single-stranded viral RNAs by DCLs [15, 20, 21]. We speculated that more frequent cleavage of transcripts of virion-sense strands might be one reason for the asymmetric distribution along the MSRV-YN genome.

**Fig. 5:**
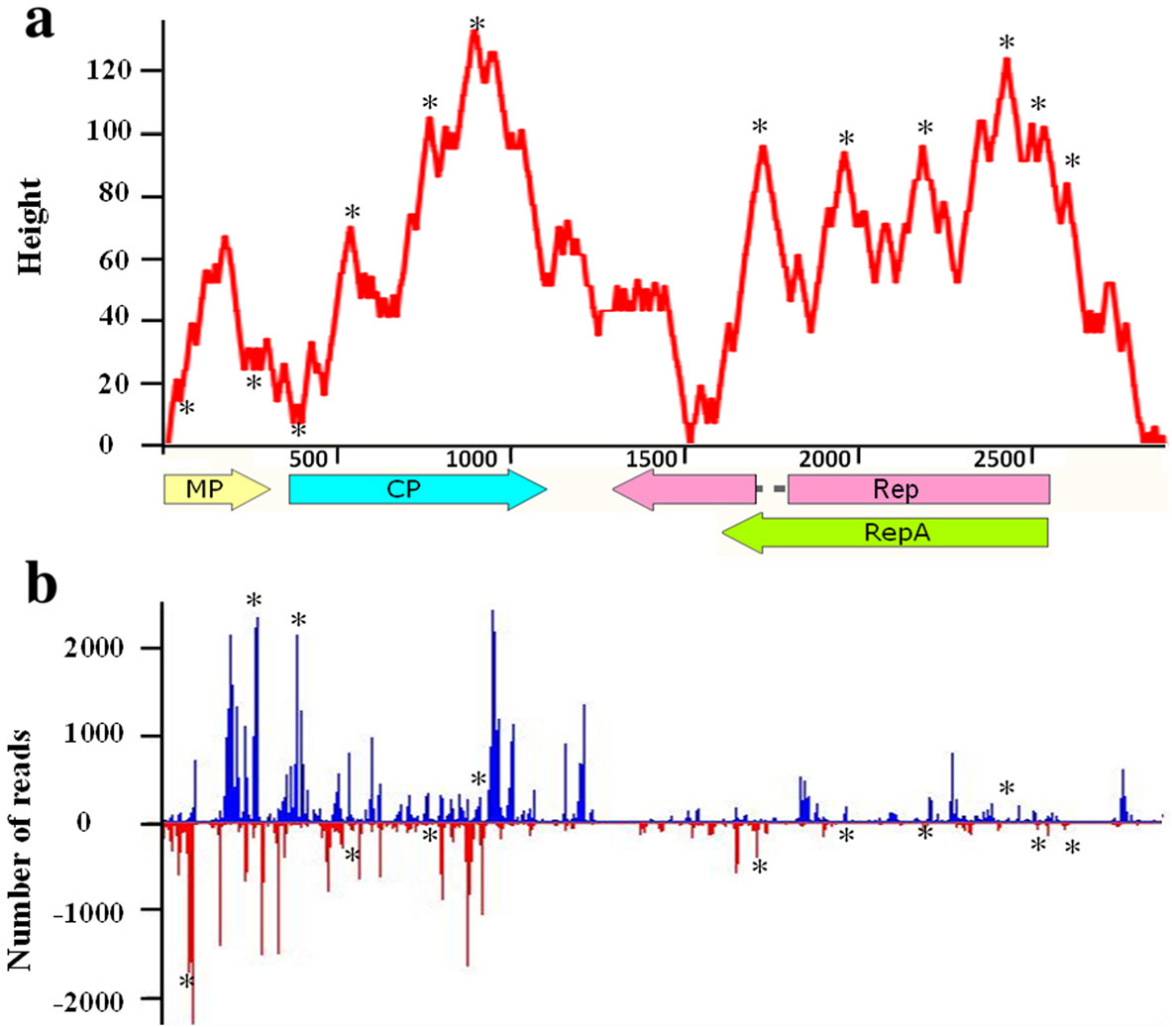
Structural analysis of MSRV-YN vsiRNA hotspots. **a** The secondary structures were predicted using the thermodynamic prediction of minimal free energy (MFE). A mountain plot representation of the MFE structure is shown. **b** Profile of the MSRV-YN-derived siRNAs. An asterisk indicates that vsiRNA production was consistent with the predicted highly structured regions. Positions and orientations of the four predicted ORFs are shown by colored arrows

A genome-wide view of vsiRNAs revealed that the vsiRNAs of MSRV-YN were not uniformly distributed along each genome segment, with the most abundant siRNAs matched both positive and negative strands of the MP and CP-coding region (Fig. 5), indicating the accessibility of target sites with MP and CP. The peaks of hotspots were particularly observed in the position of 75 to 385 nt and 882 to 954 nt along MSRV-YN genome. Hotspots for vsiRNAs generation have been extensively described and discussed for many viruses, yet the reasons for this phenomenon are not entirely clear [22–25]. Previous reports indicated that imperfect duplexes in the most-folded regions might be targeted for specific DCLs cleavage and thus affect vsiRNAs production [24]. On the other hand, evidence showed that a hairpin structure of *Cucumber mosaic virus* satellite RNA could be targeted more efficiently by DCL4 [26]. Since MSRV-YN is a DNA virus, we inferred that the vsiRNAs might be predominantly produced by DCL cleavage of highly structured transcripts. To get a clearer insight into the relationship between secondary structure and hotspots generation, we analyzed the putative secondary structures formed by MSRV-YN transcripts using RNAfold [27]. A great degree of coincidence between secondary structures and hotspot generation was demonstrated as expected (Fig. 5), indicating that the secondary structures of the MSRV-YN transcripts contributed greatly to the production of viral siRNAs. Yet, we cannot rule out that other reasons contribute to the greater suitability of certain regions for the formation of siRNAs. More work is still needed to understand this asymmetry of siRNAs generated from different region of viral genome.

## Conclusion

Currently, mastreviruses are receiving more and more attention since they have been associated with many serious crop diseases. Of the 29 currently recognized mastrevirus species, 23 include viruses that can infect monocotyledonous hosts and 6 include viruses that infect dicotyledonous hosts [28]. To date, other than MSV, MSRV is the only mastrevirus species that has ever been sampled from maize having maize streak disease symptoms. Interestingly, MSRV was also detected from wild grasses such as *Setaria barbata* and *Rottboellia* sp. in Nigeria, suggesting expanded host and geographical ranges for this virus [5]. This first report of MSRV isolates in China reveals that this virus is likely to possess a far greater diversity and distribution than has been appreciated. Because 10 of 22 samples from Yunnan Province, China, were infected with MSRV-YN, for an infection rate of 45.5 %, further work on epidemics of MSRV-YN in China is needed.

Recent advances in deep sequencing have greatly accelerated the accuracy and rate of virus discovery. Our study, the first to use deep sequencing to characterize siRNAs derived from MSRV-YN, should provide guidelines on designing antiviral strategies using RNAi against MSRV-YN.

## Materials and methods

### Samples collection and total RNA extraction

In May 2014, 22 maize (*Zea mays*) leaf samples were collected from five maize fields in Yuanmou County, Yunnan Province, China, the distance between which ranged from 100 m to 2000 m more than. These samples displaying a range of disease symptoms such as yellowing and chlorotic streaks along leaf veins (data not shown) were stored at −80 °C. Total RNA was extracted from 0.1 g of symptomatic maize leaf according to the manufacturer’s instructions (Invitrogen, Carlsbad, USA). The purified RNA preparations were evaluated using a spectrophotometer (Nanodrop, Thermo Fisher Scientific, Waltham, MA, USA) and agarose gel electrophoresis. The better quality RNA described as YM was shipped with dry ice to the Beijing Genomics Institute (BGI) for small RNA library construction and sequencing.

### Small RNA deep sequencing and data processing

Sequencing was performed according to the manufacturers’ protocol by using the high-throughput Illumina HiSeq-2000 sequencing technology. Data processing was conducted using a custom bioinformatics pipeline. Raw data were analyzed using an in-house Perl script, and 18–28 nt reads consisting of trimmed sRNA sequences were collected for subsequent analysis. The Velvet program [29] was used for genome assembly and a parameter of 17 nucleotides was set as the minimal overlapping length (*k*-mer) required for joining two sRNAs into a contig [9]. The assembled contigs were then aligned with the nonredundant (nr) nucleotide sequences from the National Center for Biotechnology Information (NCBI) database using BLASTn (nucleotide blast) with standard parameters [9].

### Total DNA extraction and cloning of full-length viral genome

A CTAB-based extraction method [30] was used to obtain total DNA from 0.1 g maize leaves sample. The full-length viral genome was acquired by PCR amplification using a pair of adjacent primers F/R (Additional file 1 Table S1), which were designed based on the assembled contigs. The resulting PCR products were then cloned into vectors using ZeroBack Fast Ligation Kit (TIANGEN BIOTECH CO.) and sequenced by conventional Sanger dideoxy sequencing. The DNAStar Lasergene package (Version 7.1.0) was used for full-length nucleotide sequences assembly of the viral genome.

### Detection of MSRV-YN by rolling circle amplification (RCA) and Southern blot

The rolling circle amplification of circular DNA was conducted using TempliPh 500 Amplification Kit (GE Healthcare) according to the manufacturer’s protocol. From 10 to 100 ng of total deoxyribonucleic acids was dissolved in 5 μl sample buffer, denatured at 95 °C for 3 min and then immediately cooled down on ice for 10 min. Subsequently, 5 μl reaction buffer and 0.1 μl enzyme mix were added, and the reaction was run at 30 °C for 18 h. The reaction was then stopped at 65 °C for 5 min. The RCA products were digested with the single restriction enzyme BamHI, and fragments were then separated on 1 % agarose gels, excised and recovered using a Gel Extraction Kit (Axygen Scientific Inc). For Southern blot analysis, 20 μg of total DNA preparations and 20 ng RCA recovered products were used and hybridized with a virus-specific probe according to standard Southern blot protocols. The probe was synthesized using the primer pair F1/R1 (Additional file 1: Table S1) and digoxigenin-labelled (Roche, DIG-High Prime DNA Labeling and Detection Starter Kit) for chromogenic substrate detection with alkaline phosphatase (AP) after hybridization according to the manufacturer’s instructions.

### Nucleotide sequence analysis and phylogenetic analysis

Open reading frames (ORFs) were predicted using the ORF finder function of the Snap Gene software [10]. Nucleotide and amino acid identities were computed using the MegAlign software (Version 7.1.0). Phylogenetic trees were built with the neighbor-joining (NJ) algorithm within the Molecular Evolutionary Genetics Analysis program package (MEGA 6) [13]. Multiple alignments of nucleotide sequences were conducted using Clustal W [12]. Amino acid sequences were aligned using the MUSCLE algorithm [31]. The reliability of each branch was evaluated with a bootstrap of 1,000 replicates. Other parameters were set to the default value. Selected sequences used in the phylogenetic analysis are listed in supplementary information (Additional file 1: Table S3).

### Viral derived siRNA analysis

Small RNA mapping was conducted by Bowtie 1.0 with zero mismatch, and results were exported to Microsoft Excel for further analysis. The program MISIS [32] was used to analyze maps of small RNAs derived from viruses and genomic loci generating multiple small RNAs. Structural analysis of viral vsiRNA hot spots in viral genome was carried out using RNAfold software [27]. Secondary structures of RNAs were predicted using the thermodynamic prediction of minimal free energy (MFE) [33].

## Additional file

Additional file 1: Table S1. Primers used for PCR amplification. **Table S2.** Contigs found to have identity to *Maize streak Reunion virus* in a BLASTn search of the NCBI data base. **Table S3.** Sequences of selected members belonging to the *Geminiviridae* used in the phylogenetic analysis. (DOC 75 kb)
