# Supplementary material for: Identification and characterization of a maize-associated mastrevirus in China by deep sequencing small RNA populations
Source: Virol J. 2015 Oct 5;12:156. doi: 10.1186/s12985-015-0384-3 (PMC4594918; doi:10.1186/s12985-015-0384-3)
Supplement: Additional file 1: Table S1. — Primers used for PCR amplification. Table S2. Contigs found to have identity to Maize streak Reunion virus in a BLASTn search of the NCBI data base. Table S3. Sequences of selected members belonging to the Geminiviridae used in the phylogenetic analysis. (DOC 75 kb) [file 12985_2015_384_MOESM1_ESM.doc]

**Supplementary Table 1.** Primers used for PCR amplification

| Name | Sequence (5′→3′) |
| --- | --- |
| F | CTTGAGCCTAGTAACCCAGTCT |
| R | GGCTGCGGGTATACCTGCAA |
| F1 | TTACCAACGGAAGCTCAAGCA |
| R1 | AGCGAAGATGTCCTTCAACGTA |

**Supplementary Table 2.** Contigs found to have identity o *Maize streak Reunion virus* in a BLASTn search of the NCBI datanase

| Contig name | Length | Identity | Strand |
| --- | --- | --- | --- |
| NODE_2 | 67 | 67/67 (100%) | Plus/Plus |
| NODE_28 | 86 | 86/86 (100%) | Plus/Minus |
| NODE_47 | 352 | 339/352 (96%) | Plus/Plus |

NODE_2:

CTGTCTTATCGGTGGTTCATCAGAGATGTCATCATACTGCTTAGGGCGAAGAGACAGCGGAGCACGG

NODE_28:

ACGGAACCACGGATCGCCGGCGGGGGGTGCCGGCGGAGACTGGGTTACTAGGCTCAAGGGCTGCGGGTATACCTGCAACGTCGATG

NODE_47

CGAGACTATGACGTATAAAATAGCGTTAGATCTGCATTTCAGTATTACTGCTGAAGCCTCTGCTTATAGCAATTCAGGTACAGGGGTCTTGTGGTTAATTTACGATTCGCAGCCGAACGGAGCTCAGCCTACGTTGAAGGACATCTTCGCTTACGAGGATTCACTGGTAGCGTGGCCTTACACCTGGAAGGTCTCAAGAGAGGTCTGTCATCGCTTTGTGGTTAAACGGAGGTATACGTTCACACTCGAGTCCAATGGAAGAAGGGCCGATGAGAAGCCGCCAGCAAACTCAGTTTGGCCGCCTTGTAAGACGCACGTGTACTTCCACAAGTTCGCTAAGGGTTTGGGCGTG

**Supplementary Table 3.** Sequences of selected members belonging to the *Geminiviridae* used in the phylogenetic analysis

| Genus | Virus | Abbreviation | GenBank accession |
| --- | --- | --- | --- |
| *Begomovirus* | *Tomato leaf curl virus* | ToLCV | U88692 |
| *Begomovirus* | *Tomato yellow leaf curl China virus* | TYLCCNV | NC_004044 |
| *Begomovirus* | *Tobacco curly shoot virus* | TbCSV | JX457342 |
| *Begomovirus* | *Ageratum yellow vein virus* | AYVV | NC_004626 |
| *Begomovirus* | *Tomato yellow leaf curl virus* | TYLCV | EU085423 |
| *Begomovirus* | *Cotton leaf curl Multan virus* | CLCuMV | KP762786 |
| *Topocuvirus* | *Tomato pseudo-curly top virus* | TPCTV | NC_003825 |
| *Turncurtovirus* | *Turnip curly top virus* | TCTV | NC_014324 |
| *Becurtovirus* | *Spinach curly top Arizona virus* | SCTAV | NC_015051 |
| *Becurtovirus* | *Beet curly top Iran virus* | BCTIV | JQ707944 |
| *Curtovirus* | *Beet curly top virus* | BCTV | NC_001412 |
| *Curtovirus* | *Spinach severe curly top virus* | SSCTV | NC_014631 |
| *Curtovirus* | *Horseradish curly top virus* | HrCTV | NC_002543 |
| *Eragrovirus* | *Eragrostis curvula streak virus* | ECSV | NC_012664 |
| *Mastrevirus* | *Wheat dwarf virus* | WDV | HF968638 |
| *Mastrevirus* | *Oat dwarf virus* | ODV | NC_010799 |
| *Mastrevirus* | *Eragrostis minor streak virus* | EMSV | NC_015553 |
| *Mastrevirus* | *Chickpea yellows mastrevirus* | CpYV | JN989439 |
| *Mastrevirus* | *Chickpea chlorosis virus* | CpCV | KC172700 |
| *Mastrevirus* | *Tobacco yellow dwarf virus* | TobYDV | NC_003822 |
| *Mastrevirus* | *Paspalum dilatatum striate mosaic virus* | PDSMV | NC_018576 |
| *Mastrevirus* | *Paspalum striate mosaic virus* | PSMV | NC_018530 |
| *Mastrevirus* | *Bromus catharticus striate mosaic virus* | BrSMV | NC_014822 |
| *Mastrevirus* | *Digitaria streak virus* | DSV | NC_001478 |
| *Mastrevirus* | *Panicum streak virus* | PanSV | GQ415392 |
| *Mastrevirus* | *Wheat dwarf India virus* | WDIV | KJ028209 |
| *Mastrevirus* | *Maize streak Reunion virus* | MSRV_NG | KJ437669 |
| *Mastrevirus* | *Maize streak Reunion virus* | MSRV_NG | KJ437670 |
| *Mastrevirus* | *Maize streak Reunion virus* | MSRV | JQ624879 |
| *Mastrevirus* | *Maize streak Reunion virus* | MSRV | NC_017917 |
| *Mastrevirus* | *Maize streak Reunion virus* | MSRV | JQ624880 |
